# PReS-FINAL-2166: Long-term safety and effectiveness of anti-interleukin-6 receptor monoclonal antibody, tocilizumab, in patients with systemic juvenile idiopathic arthritis in Japan

**DOI:** 10.1186/1546-0096-11-S2-P178

**Published:** 2013-12-05

**Authors:** S Yokota, T Nozawa, T Kanetaka, K Yamazaki, T Sato, N Sakurai, M Kikuchi

**Affiliations:** 1 Department of Pediatrics, Yokohama City University School of Medicine, Yokohama, Japan

## Introduction

Systemic-onset juvenile idiopathic arthritis (sjia) is a form of childhood chronic arthritis of unknown etiology with systemic manifestations such as remittent fever and erythematous rash, lymph adenopathy, hepatosplenomegaly, and/or serositis. Tocilizumab (TCZ) is a humanized anti-IL-6 receptor monoclonal antibody that has been approved for the treatment of patients with sjia. Results of the lead-in phase; the placebo-controlled, double-blind phase; and the first 48 weeks of an open-label extension have been already published.

## Objectives

To assess the long-term safety and effecacy of tocilizumab in sjia.

## Methods

The long-term extension phase of two pivotal studies (Phase II study with 11 patients and Phase III study with 56 patients) in patients with active sjia was analyzed. Patients received open-label tocilizumab (8 mg/kg, every 2 wks). Assessments included ACR Pedi 30/50/70 responses, adverse events (aes), laboratory parameters, oral corticosteroid dose reductions, and growth rates (the latter compared with national standards).

## Results

In total, 67 patients were enrolled. Median duration of exposure was 3.4 years. Event rates of aes and serious aes were 803.7/100 patient-years (pys) and 34.7/100 pys, respectively. The most common serious aes were infections (13.2/100 pys). Mean liver enzyme levels basically remained stable over the course of the study. Grade 3 elevations in alanine and aspartate aminotransferase levels occurred in six (9.0%) and four (6.0%) patients, respectively. Mean total cholesterol levels also were not increased during the study, although eight (11.9%) patients experienced grade 2 elevations in total cholesterol. ACR Pedi response rates were maintained throughout the study: at week 168, ACR Pedi 30/50/70 response rates were 80.3%, 80.3%, and 75.4%, respectively. Tocilizumab completely blocked the production of C-reactive protein and serum amyloid A in sjia. Twenty-two (32.8%) patients discontinued corticosteroids during tocilizumab therapy. Of 52 patients completing the week 168 visit, 40 (76.9%) reduced corticosteroid doses by at least 50%, and 33 (63.5%) patients by 70%. Significant improvement was seen in changes in height velocity (HV) SDS from 1 year prior to 1 year after baseline. HV-SDS continued to improve through the studies of toiclizumab treatment.

## Conclusion

Tocilizumab had an acceptable safety profile, was associated with sustained clinical improvement, and reduced systemic corticosteroid dose in children with sjia. Also, catch-up growth was observed in patients under the treatment with tocilizumab.

## Disclosure of interest

S. Yokota Consultant for: an advisory board from Chugai Pharmaceuticals; joint patent holder for tocilizumab for treatment of systemic juvenile idiopathic arthritis., T. Nozawa: None declared., T. Kanetaka: None declared., K. Yamazaki: None declared., T. Sato: None declared., N. Sakurai: None declared., M. Kikuchi: None declared.

